# Fluid balance of female para hockey players during simulated competition

**DOI:** 10.3389/fspor.2024.1479879

**Published:** 2024-11-25

**Authors:** Erica H. Gavel, Samantha Rae, Heather M. Logan-Sprenger

**Affiliations:** ^1^ Faculty of Science, Ontario Tech University, Oshawa, ON, Canada; ^2^ Department of Family Medicine and Physical Medicine and Rehabilitation, University of Michigan, Ann Arbor, MI, United States; ^3^ Tanenbaum Institute for Science in Sport, University of Toronto, Toronto, ON, Canada; ^4^ Faculty of Health Science, Ontario Tech University, Oshawa, ON, Canada; ^5^ Faculty of Science and Faculty of Health Science, Ontario Tech University, Oshawa, ON, Canada

**Keywords:** sledge hockey, para ice hockey, hydration, impairment, performance

## Abstract

**Purpose:**

The purpose of this study was to characterize the hydration habits and fluid balance of female para-ice hockey players.

**Methods:**

Fifteen players [5 defense (D), 8 forwards (F), and 2 goalies (G)] volunteered to participate in the study (age: 26.3 ± 10.9 y; ht:155 ± 11 cm; arm length: 65 ± 8 cm; leg length: 88 ± 11 cm; trunk length: 66 ± 14 cm). Players were weighed pre- and postgame, while fluid intake and individual playing time (PT) was monitored throughout the game.

**Results:**

On average, athletes arrived hydrated to the game (USG 1.019 ± 0.006) with 40% of players arriving dehydrated (USG >1.020). Mean playing time for forwards was 11:47–28:49 min:s (18:52 ± 5:48 min:s) and for defence 13:10–18:24 min:s (15:10 ± 2.05 min:s). Sweat loss was 0.96 ± 0.64 L (0.10–2.50 L) which exceeded net fluid intake (0.61 ± 0.37 L). Mean BM loss was 0.44 ± 0.9% (−2.1 to +0.9%) with 4 of 15 players (2 D, 1 F, 1 G) losing between 1.4 and 2.1% BM. Players preferred to drink water during the game compared to a carbohydrate electrolyte solution.

**Conclusion:**

60% of athletes arrived hydrated to the game and drank enough fluid to prevent a BM loss <1%. Of note is that 40% of players arrived at the arena mildly dehydrated based on USG, and despite abundant opportunities to drink fluid during the game, 25% of players lost >1.3% BM due to sweat loss which may compromise physical and cognitive performance.

## Introduction

Para ice hockey, previously known as sledge hockey, is an adaptive version of able-bodied ice hockey with similar game rules (1). In para ice hockey, athletes are in a seated position on an aluminum sledge with two blades attached and straps securing their hips, knees, and ankles. Each athlete carries two sticks, one in each hand, for puck handling and maneuvering on each side of the sledge (1). Excluding the skates and sticks, the rest of the equipment worn by the athletes is standard ice hockey equipment (2). In terms of ice surface, the game is played on a 60 × 30 m ice rink and consists of three 15-minute periods, with two 15-minute intermissions between periods (1). Para ice hockey is characterized by repeated high-intensity short sprints (6–22 m) relying mainly on the athletes’ upper body for propulsion (2) with each shift lasting approximately 3–4 min (1, 3). While the team consists of individuals with varying impairments that generally affect their lower body (2), the most common impairments include amputations, neuromuscular impairments, and congenital anomalies (2). Work by Molik et al. (4) studying disability and anthropometric variables in elite sledge hockey players participating in the 2010 Winter Paralympic Games reported leg amputation being the most common with a total of 27 of 54 (50%) participants, followed by spinal cord injuries (SCI) with 17 (31%). Furthermore, Baumgart and Sandbakk (1) reported similar results with the most common impairment in para ice hockey being leg amputation and spina bifida.

Similar to able-bodied ice hockey, para ice hockey is played in a cool environment (∼10°C) with multiple layers of equipment worn, which creates a microenvironment between the equipment and the skin, limiting access for convective cooling, impacting the athlete's heat exchange with the environment (5–7). As well, Emerson et al. (8) demonstrated that training in a cool environment impaired athletes’ thirst response and lead to lower fluid intake (8). Work by Logan-Sprenger et al. (5) in able-bodied junior male hockey players demonstrated high mean sweat losses (3.2 ± 0.2 L) which exceeded fluid intake (2.1 ± 0.1 L), with 8/24 players losing between 1.8%–4.3% BM loss. More recent work by Driscoll et al. (9) examining fluid vs. no-fluid effects on varsity level women's able-bodied simulated game performance, reported that mild dehydration of 1.7 ± 0.3% BM loss resulted in significantly higher core temperature, perceived fatigue and lower sprint power performance. Of interest, is research published by Bigg et al. (7) which reported that 24 female varsity ice hockey players monitored over four games demonstrated a mean sweat loss of 1.01 ± 0.29 L and 0.70 ± 0.43 L of fluid intake resulting in minimal BM losses (<1% BM for all players). Meanwhile, ∼50% of athletes arrived to the game mildly dehydrated (USG >1.020). The adverse impact of mild dehydration (1.5%–2% body mass loss) on athletic performance—particularly reductions in sprint capacity and increased perceived fatigue—has been extensively studied in able-bodied ice hockey players, primarily among males. However, no research to date has explored the hydration practices or sweat loss profiles specific to female para ice hockey athletes.

The impact of mild dehydration (∼2% BM loss) on performance across exercise and sport in able-bodied athletes has been well studied (5, 6, 10–15). In contrast, there are very few studies that have examined hydration habits and fluid balance in para-athletes in the field-of play (16, 17). The physiological and functional differences between various impairments seen in para ice hockey may influence the athletes’ ability to thermoregulate to varying degrees. For example, athletes with SCIs have an altered sudomotor function and vasomotor control below the injury site, resulting in inefficiencies in sweating and blood-flow redistribution (18, 19). Likewise, individuals with amputations have a decreased surface area, which may impact the ability to dissipate metabolic heat effectively (18). Of note, is the fact that some athletes with limited functional ability may voluntarily restrict fluid intake before and during exercise to prevent the possibility of micturition if facilities are non-accessible (20). As of late, the habitual hydration behaviors and sweat loss of para ice hockey players is unknown. The purpose of this study was to characterize habitual hydration habits and sweat loss in elite female para ice hockey players during a game.

## Methods

### Athlete characteristics

Fifteen (SCI = 4, spina bifida = 2, amputation = 2, cerebral palsy = 2, proximal femoral deficiency = 1, Perthes = 1, anthroponoses = 1, Brown Sequard syndrome = 1, Table 1) elite female para ice hockey players volunteered to participate in the study. Athletes were members of a women's para hockey team. Data collection occurred over the course of two exhibition games against the same team separated by 24 h. Athletes were separated into two separate groups and tested once—this occurred in either game one or two. All participants were informed of the experimental protocol before providing both oral and written consent to participant. Parental consent was obtained for participants under 18 years of age (*n* = 3). The study was approved by the Research Ethics Board at Ontario Tech University (Oshawa, Ont., Canada) and conformed to the principles defined in the Declaration of Helsinki.

**Table 1:** Number of shifts played, playing time (mm:ss), heart rate and rating of perceived exertion response of the female para-ice athletes during the game.

Position	Number of shifts			Playing time (min)			Mean heart rate response (bpm)			Mean rating of perceived exertion (AU)		
	P1	P2	P3	P1	P2	P3	P1	P2	P3	P1	P2	P3
Defense												
	4	3	2	04:21	04:32	4:51	137	129	144	4	4	2
	1	4	6	05:05	03:59	04:06	121	119	128	4	4.5	4.2
	3	4	4	05:34	04:29	04:10	141	139	148	4.7	4.7	5.3
	5	5	4	05:20	04:22	08:42	153	161	113	5.4	4.6	6.5
Forward												
	2	2	3	03:51	04:28	03:28	160	149	173	2	1.5	5.5
	4	3	3	12:03	04:14	04:36	88	95	97	2.7	3	2
	2	2	2	04:21	03:26	06:14	162	161	142	7.3	7	7.3
	3	2	3	12:05	03:59	04:45	136	141	138	7.3	5.7	5
	5	3	4	09:08	08:57	10:43	140	158	147	6	7	6.5
	3	3	4	04:42	05:03	05:26	162	165	175	6	6	3.7
	4	2	3	04:43	4:26	6:06	149	141	143	7.3	7	6.7
	5	4	5	12:05	05:46	06:23	153	155	149	1.2	1.5	1.2
Goalie												
	1	1	1	15:00	15:00	15:00	131	134	117	3	2	3
	1	1	1	15:00	15:00	15:00	126	126	126	3	2	3
Mean	3.1	2.8	3.2	07:55	06:08	07:01	138	140	136	5	4	4
SD	1.1	1.1	1.4	04:10	03:49	03:44	21	20	24	2	2	2

P1, period 1; P2, period 2; P3, period 3; secs, seconds; L, lumbar; T, thoracic.

### Study design and game protocol

Athletes arrived at the arena at their usual pregame time (∼2–2.5 h) and were asked to follow their typical routine prior to the game. Upon arrival, the participants voided their bladder and provided a mid-stream urine sample to measure USG using a calibrated handheld refractometer (Atago–PEN-PRO, Geneq, Montreal QC). A hydrated state exemplified a USG <1.020, with a USG >1.020 indicating a dehydrated state (5). BM was measured 45 min before the start of warm-up on a portable digital scale (Zenith LG Electronics Canada, Mississauga, Ont., Canada) with participants wearing a dry sports bra and shorts. Following the game, athletes dried themselves off with a personal towel and put the original dry clothes back on to be weighed again. All athletes were equipped with a Polar OH1 HR sensor (Polar, Canada) and heart rate response was monitored from the start to end of each period. Both games followed the same pregame routine and started at 19:30 h.

During the game and intermissions, athletes were instructed to consume *ad libitum* water and carbohydrate electrolyte solution (6% CES) as normally done during games. Water and CES (Gatorade 6% CES, PepsiCo Canada, ULC, Peterborough, Ont., Canada) were the beverages available pre-game, on the bench, and in the dressing room. The CES consisted of the following per 591 ml: sodium, 270 mg; potassium, 75 mg; carbohydrate, 36 g. The participant's bottle(s) were labeled with their names for collection along with being weighed with the initial volume of fluid. Playing time was video recorded for each athlete using a 5th generation iPad (Apple, California, USA). Playing time was characterized by the game time avoid of stoppages. The athletes 0–10 Borg Ratings of Perceived Exertion (RPE) (21) was collected after each on-ice shift and recorded by the team physical therapist, who was located on the team bench. Drink bottles were collected and measured pre-game, following warm-up, between each period, and post-game to determine the total amount of fluid consumed by each athlete. The arena temperature and relative humidity for both games were measured using a heat stress tracker [Kestral 5400; Nielsen-Kellerman, Boothwyn, PA; accuracy, ±0.5°C, ±2% relative humidity (RH), wind speed ±3%]. Post-game urine volume and BM was measured to calculate sweat loss. Heart rate was continuously collected with a Polar OH1 HR sensor (Polar, Canada).

### Calculations

1. Percentage of BM loss during the game was estimated as the net BM loss (kg) during the game divided by the pre-game BM (5)

%BM loss = (pre-BM—post-BM)/(pre-BM) × 100

2. Sweat loss (L) was estimated as the net BM loss (kg) during the game (assuming 1 kg = 1 L) minus any urine production produced (L) during the game (5)

Sweat loss = (pre-BM—post-BM) + fluid intake—urine output

### Statistical analysis

All data was tested for normality of distribution and displayed as the mean and standard deviation; where pertinent, the min-max range was included in parentheses. Differences between periods was analyzed using a one-way ANOVA, and time verses group was tested using a mixed-ANOVA and 2 × 2 ANOVA, to detect singular differences, and a Tukey's significant difference (HSD) post-hoc was performed. A student's paired *t*-test was used to compare singular parameter differences where appropriate. Categorical data was tested using the Kruskal-Wallis test, to detect singular differences a Dunn's post-hoc test was performed. Statistical significance was accepted at *p* < 0.05. Correlations between variables were assessed using a Pearson's correlation analysis. Exact *p*-values, Cohen's D, and 95% confidence intervals are presented to show magnitude of effect. The magnitude of effect was classed as trivial (<0.2), small (0.2–0.6) moderate (0.6–1.2), large (1.2–2.0), and very large (≥2.0) (22). Two forwards were removed from the sweat loss, fluid intake, and %BM loss data due to inability to be weighed pre- and post-game.

## Results

### Ambient conditions

The mean game temperature and relative humidity was 15.1 ± 0.3°C and 50 ± 1%.

### Urine specific gravity

**Figure 1:**
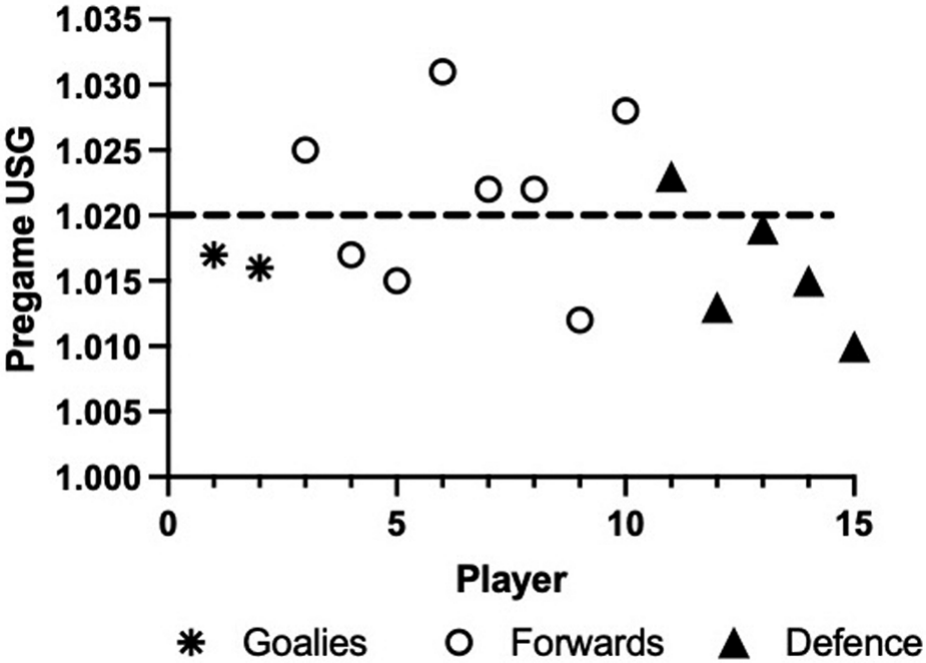
Pregame hydration [urine specific gravity (USG)] status. Individual data are presented (*n* = 15).

A pregame urine sample revealed that on average, athletes arrived at the game in a hydrated state (USG 1.019 ± 0.006). Mean USG for defense was 1.016 ± 0.005, for forwards 1.022 ± 0.007, and for goalies 1.017 ± 0.0001 (Figure 1). Of the 15 players, 40% (1/5 defense and 5/8 forwards) arrived at the game dehydrated (USG 1.031, 1.028, 1.025, 1.023, 1.022, and 1.022).

### Game playing time

There was large variability in playing time between and within positions. Forwards ranged from 11:47 to 28:49 min:s (18:52 ± 5:48 min:s) and defense 13:10–18:24 min:s (15:10 ± 2:05 min:s). The selected goalie for each game played the entirety of the match (45 min). Average playing time per period with 6:49 ± 3:15 min:s for period 1 (F 7:52 ± 3:50 and D 5:09 ± 0:30), 4:46 ± 1:22 min:s for period 2 (F 5:02 ± 1:43 and D 4:20 ± 0:13 min:s), and 5:47 ± 1:59 min:s for period 3 (F 5:47 ± 1:59 and D 5:31 ± 1:54 min:s). On average, forwards played 41% of the game and defense played 34% of the game.

### Sweat loss and fluid intake

**Figure 2:**
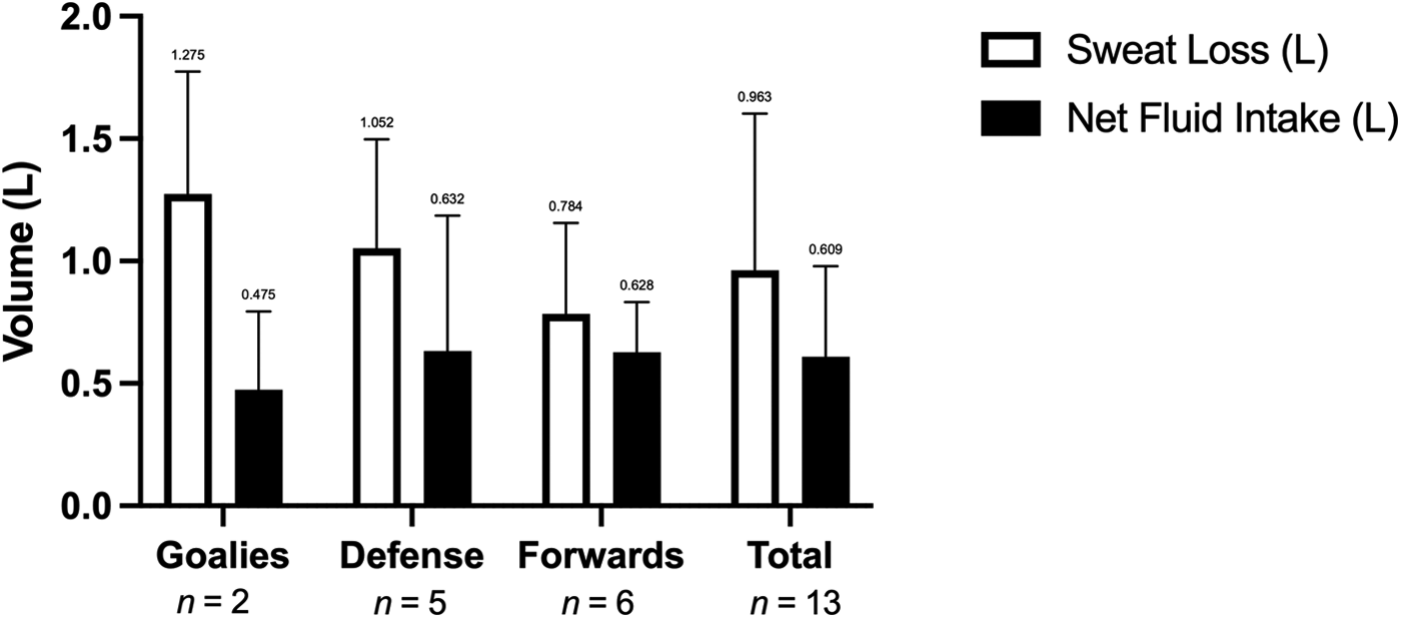
Net fluid intake (L) and sweat loss (L) during a hockey game. Data are presented as mean ± SD, *n* = 15.

**Figure 3:**
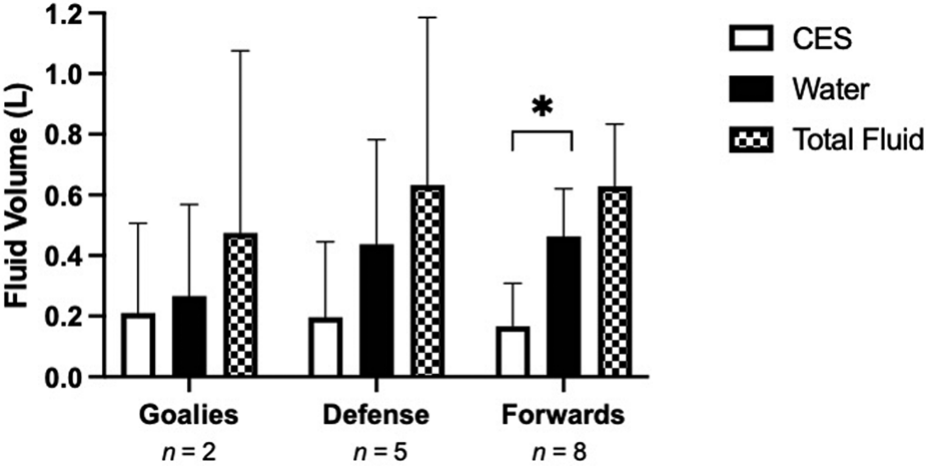
Mean water, carbohydrate electrolyte solution (CES), and total fluid intake during the game and for each position. *p < 0.001 water vs. CES. Data are presented as means ± SD, *n* = 15.

Mean total sweat loss during the game was 0.96 ± 0.64 L (0.10–2.50 L, Figure 2), with a mean rate of 1.26 ± 0.89 L/hr. Mean fluid intake was 0.61 ± 0.37 L and ranged from 0.10–1.35 L, with a significantly greater consumption of water (0.43 ± 0.24 L (0.1–0.76 L) vs. CES (0.19 ± 0.19 L (0–0.42 L) (*p* = 0.0014) for all positions (Figure 3). The mean fluid intake rate was 0.92 ± 0.66 L/hr. No player micturated during the game. Players voluntarily replaced 81 ± 47% (0%–140%) of sweat losses during the game with large variability between players.

Despite the same opponent for both games and level of play, the 2 goalies had very different sweat losses (0.1 and 2.5 L respectively) and replaced 68 ± 45% of sweat loss. The defense averaged a sweat loss of 1.05 ± 0.46 L and replaced 64 ± 47% of sweat lost, while forwards lost 0.78 ± 0.37 and replaced an average of 107 ± 51%.

### Body mass loss

Players on average lost 0.44 ± 0.9% BM by the end of the game (range, −2.1–0.9%). Mean BM loss was 0.61 ± 0.8 for the defense, 0.1 ± 0.8 for the forwards, and 1.0 ± 1.5% for the goalies. Of all the players, 4/13 (31%) lost between 1.4% and 2.1% BM.

### Heart rate response during the game

**Figure 4:**
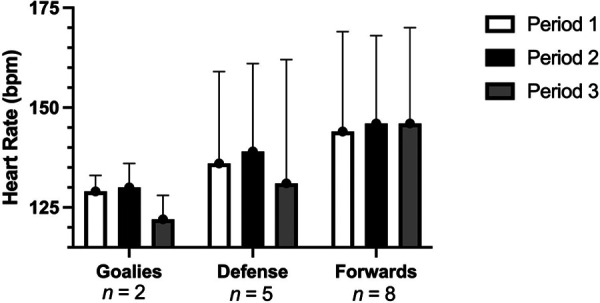
Mean heart rate response during the game for each position. Data are presented as means ± SD, *n* = 15.

Average heart rate across the game was 139 ± 22 bpm for all positions. The defense average game HR response was 135 ± 23 bpm [Period (P)1: 136 ± 23, P2: 139 ± 22; P3: 131 ± 31, Figure 4], while forwards average game heart rate response was 145 ± 23 bpm (P1: 144 ± 25, P2: 146 ± 22, P3: 146 ± 24 bpm), and goalies averaged 127 ± 1 bpm over the game (P1: 129 ± 4, P2: 130 ± 6, and P3: 122 ± 6 bpm). There was no significant difference in HR response between periods for all players and within positions (*p* > 0.05).

### Rating of perceived exertion

Mean RPE during the game for all positions was 4.4 ± 1.9 AU (1–7 AU). Defense averaged an RPE of 4.4 ± 1 AU (P1: 4.8 ± 1, P2: 4.5 ± 1.0, P3: 3.9 ± 1.7), forwards averaged an RPE of 4.8 ± 2.2 AU (P1: 4.8 ± 2.5, P2: 4.7 ± 2.4, P3: 2.2 ± 3.0 AU), and goalies averaged 2.7 ± 0 AU (P1: 3.0 ± 0, P2: 2.0 ± 0, P3: 3.0 ± 0 AU) over the game. There was no significant difference in RPE between positions or between periods (*p* > 0.05).

### Correlations

**Figure 5:**
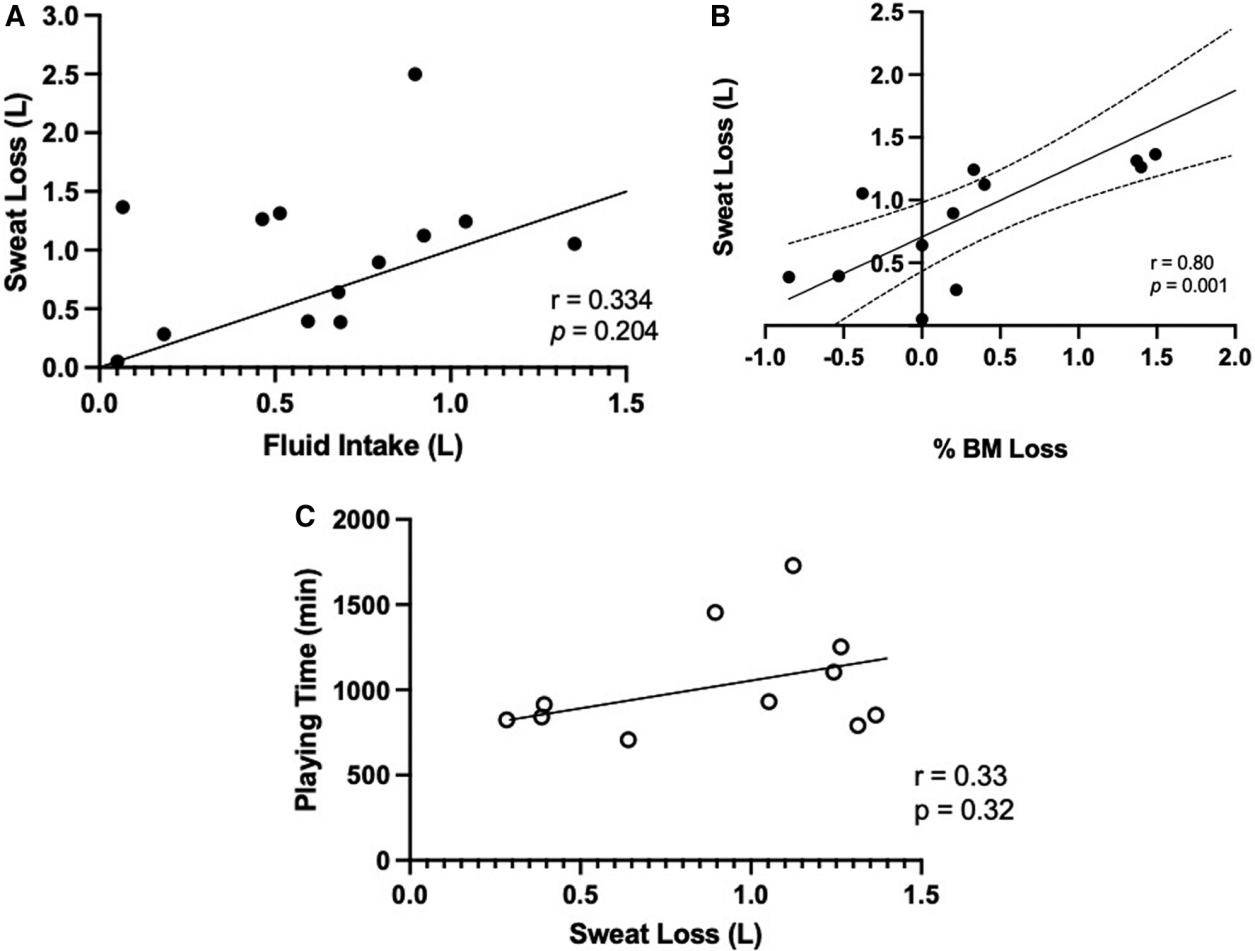
Relationship between **(A)** fluid intake (L) and sweat loss (L) over the game, and **(B)** percentage of body mass (%BM) loss and sweat loss (L), **(C)** playing time (min) of forwards and defense (*n* = 11) and sweat loss (L). Data are presented as means ± SD, *n* = 13.

Pregame USG did not correlate with net fluid intake throughout the game (*r* = 0.12, *p* = 0.69). Pregame USG was not significantly correlated to %BM loss (*r* = −0.14, *p* = 0.648). Likewise, sweat loss was not significantly correlated with net fluid intake (*r* = 0.38, *p* = 0.21, Figure 5A); however, there was a significant relationship between sweat loss and %BM loss (*r* = 0.80, *p* = 0.001, Figure 5B). There was no significant correlation between playing time and sweat loss (*r* = 0.22, *p* = 0.47) and no significant relationship between sweat loss and playing time for the forwards and defense only (goalies removed) (*r* = 0.33, *p* = 0.32, Figure 5C). There was no relationship between mean HR response and playing time (*r* = −0.13, *p* = 0.67). There was no significant relationship between mean HR response and RPE (*r* = 0.37, *p* = 0.18) and playing time and RPE (*r* = 0.018, *p* = 0.95).

## Discussion

This is the first known study to investigate the habitual hydration habits and sweat loss of elite female para ice hockey players during a game. The results of the study showed that, (1) 40% of athletes arrived at the game in a hypohydrated state (USG >1.020), (2) mean BM loss across the game from sweat loss was 0.45 ± 0.89% with only 1 athlete losing >2% BM loss (2.1%), (3) mean athlete fluid intake was 0.56 L with significantly more voluntary water consumptions vs. CES intake.

### Pre-game hydration status

This study reported a mean USG of 1.019 ± 0.006 with 40% of athletes arriving to the game in a mildly dehydrated based on USG. This closely aligns with previous findings in able-bodied elite female hockey players demonstrating that almost half of the athletes arrived at the rink mildly dehydrated (USG >1.020) (7). Although the current study includes athletes with a variety of impairments, research by Hamouti et al. (23) suggests that USG values are accurate to detect hypohydration in individuals with lower amounts of muscle mass. Thus, USG

may be a valid indicator of hydration status in this para-athlete population, which on average, has lower muscle mass. Future research is needed to verify the validity of USG as an indication of hydration status for para-athletes due to the large variance in the amount of lean body mass between athletes and classification. The USG data reinforces the importance of hydration education to decrease the prevalence of athletes arriving to competition in a mildly dehydrated state to diminish the potentially negative effects of dehydration on physical and cognitive performance.

### Sweat loss, fluid intake, and body mass loss

In this study average sweat loss was 0.92 ± 0.70 L in elite female para ice hockey players over a 45 min game. This finding is similar to able-bodied research on female hockey players, whereby Bigg et al. (7) reported sweat losses of 1.01 ± 0.29 L over the course of a varsity ice hockey game (60 min in duration), which is the only known publication evaluating habitual fluid intake and sweat loss in a female ice hockey population albeit able-bodied. When comparing sweat loss as a function of game duration, this study demonstrated a higher athlete sweat loss compared to the Bigg et al. (7) paper (1.22 ± 0.93 L/hr vs. 1.01 ± 0.29 L/hr). That said, the number of shifts, shift duration, and playing time was not reported in the Bigg et al. (7) paper which may influence the difference in sweat loss. Moreover, the number of players in the current study was 14, whereas the study by Bigg et al. (7) had 24, suggesting that the difference in sweat loss may be due to a lower number of lines and increase in total shifts. This is an interesting finding and requires more investigation, as it was speculated that sweat loss may be compromised in this population due to disability and differing physiological impairments. Likewise, in the current study, there was no significant relationship between playing time and sweat loss due primarily to large individual variability, which is aligned with research by Logan-Sprenger et al. (5) in able-bodied male hockey players, and highlights the importance of individualizing fluid replacement strategies relative to sweat loss. This is of particular importance for para-athletes with differing levels of neurological function and sweat response (24).

Fluid intake is influenced by the opportunities to drink during rest periods and informal stoppage in play (25, 26). Hockey is a sport with frequent stoppages and quick shifts allowing for an abundance of opportunities to hydrate, which was evident in this study with the number of shifts by each athlete over a period ranging from 1 to 5 per player. Although the frequency of fluid consumption was not recorded in the present or previous studies, the total volume of fluid consumed is similar to published able-bodied hockey research by Bigg et al. (7). While the current study found that the average total fluid consumption was 0.81 ± 0.49 L/hr, work by Bigg et al. (7) exhibited similar results of 0.71 ± 0.43 L/hr. Although slightly different, the discrepancy is not substantial. Overall, athletes seem to be drinking enough fluids to negate the effects of sweat loss; however, an individual approach could be seen as beneficial. Moreover, the athletes preferred water over the CES, which is aligned with work by Logan-Sprenger et al. (5). As such, trainers, coaches, and team staff can follow similar hydration guidelines used in the able-bodied population when working with female para-hockey athletes.

When exercising, hypohydration resulting in a <2% BM loss is considered tolerable, while a >2% BM loss due to sweat loss can result in an augmented HR, core temperature, RPE, and a lowered technical and endurance performance (5, 27, 28). Given that the present study found that athletes lost an average of 0.45% of their BM by the end of the game, it is not expected that game performance was negatively impacted. Relative to able-bodied hockey players, the mean BM loss reported in this study is lower than the referenced studies reporting BM changes of 1.3%, <1%, and 1.1% respectively (5, 7, 29). Given that the present study exhibited similar pre-game hydration levels relative to Logan-Sprenger et al. (5) and Bigg et al. (7), but had nine athletes with a spinal cord injury, suggests that the discrepancy may be related to the lower muscle mass in the athletes with a spinal cord injury or other types of neurological impairments (23).

### Heart rate response and RPE

Over the course of the game, athletes displayed an overall average HR of 139 bpm, with defense exhibiting 135 ± 23 bpm, forwards 145 ± 23 bpm, and goalies 127 ± 1 bpm. Relative to able-bodied work, these results significantly differ. For example, in game intensity and analysis work by Stanula et al. (30) showed that forwards showcased a mean HR of 148–158 bpm, whereas defense showcased values of 159–178 bpm. Given the difference in functional muscle mass, one could suggest that these differences are due to total muscle activation (use arms vs. legs) verses effort or actual intensity of game (31).

Although the effects of intermittent sport on HR in female para-sport athletes is limited, these results are similar to wheelchair basketball work by Logan-Sprenger and Mc Naughton (26). In the study by Logan-Sprenger and Mc Naughton (26), the mean HR for all athletes who played over 50% of the game was 134 bpm. Given that the present study and work by Logan-Sprenger and Mc Naughton (26) used athletes of similar impairments and are both intermittent in nature, suggests the HR response may be “normal” for this given population.

Moreover, this study reported an average RPE of 4.4 AU, with previous research showing strong correlations between RPE, HR, blood lactate during intermittent team sport (32). For example, in a study by Coutts et al. (32) analyzing the relationship between HR, blood lactate, and RPE in small-sided soccer games, showed the RPE can be used as a measure of global exercise intensity. However, given that disability specific research by Paulson et al. (33) showed that the relationship between RPE and HR was insignificant with a positive relationship with blood lactate, suggests that RPE may be the most valid way to monitor exercise intensity if blood lactate is not available. Future research should analyze these relationships in para hockey.

### Practical applications

The outcomes of this study provide practical insight of the hydration habits of elite female para-ice hockey players. On average, this study demonstrates that hydration habits and are more similar than different when compared to able-bodied female hockey players. This study demonstrated that female para ice hockey athletes did a good job with their habitual fluid intake to minimize body mass loss (<1%) due to sweat loss and be encouraged to focus attention on arriving to a game in a hydrated state. Likewise, individual variability in fluid intake and sweat loss calls for a customized hydration plan for each athlete. More research is needed to validate USG to track hydration status for this population since void frequency is actively restricted due to functional ability. As well, more research is needed to assess the validity of using pre- and post-game body mass changes to track sweat loss during exercise.

## Conclusion

In conclusion, 40% of the players arrived at the game mildly dehydrated (USG >1.020). Most players preferred water over CES, with fluid replacement strategies adequate to prevent BM loss of >2% in the majority of athletes (9/13). Female elite para-ice hockey players also had similar sweat loss patterns relative to able-bodied athletes, which suggests that players and coaches can take the same approach regarding hydration habits pre and during a hockey game.

## Data Availability

The datasets presented in this article are not readily available because the data is used by high level hockey players. Requests to access the datasets should be directed to erica.gavel@ontariotechu.net.
